# British Medical Association, Bristol Division

**Published:** 1982

**Authors:** Gordon Mather


### British Medical Association, Bristol Division

**Gordon Mather**

The division has had another active year thanks to the tireless secretarial work of Miss Joan Ring, assisted by Dr. John Causton and Eric Hamblett.

### Students

As in previous years, the B.M.A. has sponsored meetings of the Galenicals, the Careers Fair and lectures on insurance, medical defence and junior doctors' contracts. The final year students celebrated the end of their exams by having tea with us on the lawns of the Royal Fort: no-one fell in the pond though it was a very warm afternoon. A very large number of students have joined as associate members which gives them the journal and access to the regional organisation which is helpful in finding locums, settling problems with employers, etc.

### Meetings

The January meeting had to be postponed on account of the heavy snow, but the speaker, Mr. Anthony Goode, F.R.C.S., hopes to tell about "Man and Medicine in Space" in September. In February we met for the first time in the new Southmead centre for medical education. The illustrated talk by Dr. Robert Sellin on the Severn Barrage was fascinating as he has himself been working on the project as a civil engineer. Obviously the medical profession was interested in the future of the estuary as there was standing room only in the lecture theatre.

In March we were spell-bound by a talk given by Pat Reid, B.Sc., M.B.E., M.C., on Prisoners of War and Escape. As an escapee himself and author of *Colditz* he gave us a real insight into the problems involved, and brought along some photographs and souvenirs. Professor Harold Ellis visited us in April to give an interesting and highly amusing illustrated talk on Royal operations. Like all the meetings this year, this was well attended; the venue was the nurses' lecture room at the B.R.I. and it was preceded by the usual convivial supper.

One hundred and eighty-five members and guests came to the summer meeting. Appropriate because of the eradication of smallpox from the world, we first visited the Jenner museum and temple of vaccinia. Then some visited the unique church before touring Berkeley Castle, ending with a salmon supper in the great hall. It was a happy evening, and I was particularly glad to see so many of our young colleagues. I see the role of B.M.A. meetings as educative in the widest sense associated with social contacts between all branches of the profession.

